# Neuromechanical bistability contributes to robust and flexible behavior in a model of motor pattern generation

**DOI:** 10.1186/1471-2202-16-S1-P33

**Published:** 2015-12-18

**Authors:** David N Lyttle, Jeffrey P Gill, Kendrick M Shaw, Peter J Thomas, Hillel J Chiel

**Affiliations:** 1 Department of Biology, Case Western Reserve University, Cleveland, OH 44106, USA; 2 Department of Mathematics, Applied Mathematics, and Statistics, Case Western Reserve University, Cleveland, OH 44106, USA

## 

Motor systems must be both robust (able to generate behavior reliably despite perturbations) and flexible (able to adapt to a variable environment). How can motor systems be insensitive to external perturbations, while simultaneously retaining sensitivity to sensory input when it is necessary? One possibility is that motor systems respond to changing environmental demands by switching between different stable dynamical regimes, each having different sensitivities to sensory input and producing different behaviors (multifunctionality). Multifunctional motor systems have been studied in many species, and prior work suggests it can arise from multistable dynamics in the nervous system and/or the kinematics of the body [1,2]. Here we explore how multistability can enable a motor system to rapidly adjust its sensitivity to mechanosensory perturbations, thereby allowing it to shift the balance between robustness and flexibility as needed. Specifically, we demonstrate the coexistence of multiple stable dynamical regimes with different sensitivities to sensory inputs in a neuromechanical model, and explore the functional consequences of this multistability.

We focus on a nominal neuromechanical model of the feeding apparatus of the marine mollusk Aplysia californica as a concrete example [3]. In this model, the dynamics of three mutually inhibitory neural pools yield a stable heteroclinic channel, in which a stable limit cycle passes near some number of saddle equilibrium points, thus creating regions of localized slowing where sensory inputs can prolong specific phases of the cycle [4,5]. The neural dynamics drive a biomechanical model of the Aplysia buccal mass, and proprioceptive feedback from the feeding apparatus is in turn sent back to the nervous system. The model is confronted with environmental variability in the form of a simulated feeding task: On each behavioral cycle, the feeding apparatus attempts to grasp a length of seaweed (represented as a fixed mechanical load), but only succeeds in doing so with some probability. Once a piece of seaweed has been successfully grasped, the system must consume the entire seaweed strip before attempting to grasp another one. Overall performance is assessed by averaging the net seaweed consumption over many trials.

We find that the model exhibits bistability between two distinct regimes: a "heteroclinic regime" and a "limit cycle regime." In the heteroclinic regime, sensory feedback is able to selectively slow the neural dynamics, thus providing the (slower) muscles with sufficient time to adapt to mechanical loads. In the limit cycle regime, the dynamics are insensitive to proprioceptive feedback, and the oscillations are faster. We find that the limit cycle regime is better at grasping seaweed due to the increased frequency of cycling, whereas the heteroclinic regime performs better with respect to consuming seaweed once it has been grasped, due to the enhanced sensitivity to sensory input. Moreover, the ability of the model to flexibly transition between regimes allows it to outperform variants of the model that can only operate in one regime or the other. Thus, multistable dynamics that arise as a consequence of brain-body coupling may allow motor systems to produce robust and flexible behaviors in variable environments.
